# Structural variation drives praziquantel response and host adaptation in *Schistosoma japonicum*

**DOI:** 10.1016/j.isci.2026.116118

**Published:** 2026-05-27

**Authors:** Qi Liu, Ke Yang, Wei Zhang, Shuhua Xu, Wei Hu, Yan Lu

**Affiliations:** 1 State Key Laboratory of Genetics and Development of Complex Phenotypes, Human Phenome Institute, Zhangjiang Fudan International Innovation Center, Center for Evolutionary Biology, School of Life Sciences, Department of Liver Surgery and Transplantation, Liver Cancer Institute, Zhongshan Hospital, Fudan University, Shanghai 200433, China; 2 Ministry of Education Key Laboratory of Contemporary Anthropology, Fudan University, Shanghai 200438, China; 3 School of Life Science and Technology, ShanghaiTech University, Shanghai 201210, China; 4 College of Life Sciences, Inner Mongolia University, Hohhot 010070, China

**Keywords:** immunology, parasitology, genomics

## Abstract

Structural variation (SV) is a major yet underappreciated source of genomic diversity that drives parasite adaptation. We generated the first comprehensive SV map of *Schistosoma japonicum* from 72 genomes across six endemic Asian regions, identifying 12,632 high-confidence SVs spanning 3.55% of the genome that delineate population structure and adaptive trajectories. SVs are non-randomly distributed and preferentially affect regulatory regions rather than coding sequences. Geographic isolation, demography, and positive selection collectively shape the SV landscape. Convergent deletions in *SCNN1A*, a sodium channel gene, occur in Southeast Asian populations under long-term praziquantel (PZQ) exposure and may alter its active pocket and transport activity. Taiwan-specific SVs in *SETD4*, *GDPD1*, and *PAFAH1B1* are linked to reproductive adaptation and reduced pathogenicity. Expression assays confirm stage- and sex-specific regulation of these genes under PZQ treatment. These findings establish SVs as key drivers of drug response and host adaptation in *S. japonicum*, offering insights for precision surveillance and next-generation anthelmintic design.

## Highlights

• We generated the first structural variation map of *Schistosoma japonicum*
• Structural variations reveal fine-scale population structure across endemic regions
• Convergent deletions are associated with praziquantel response in island populations
• Highly differentiated variations are linked to reduced reproduction and virulence

## Graphical abstract

**Figure:**
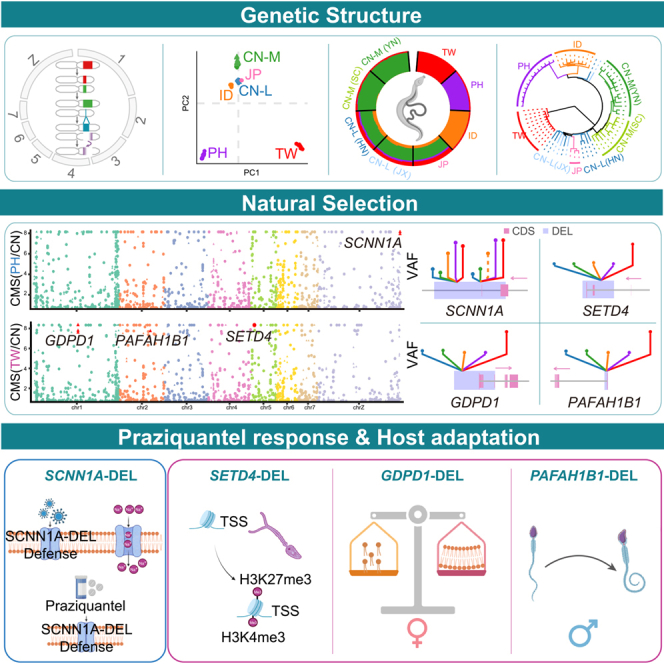

## Introduction

Schistosomiasis, the most devastating parasitic disease after malaria, remains a major global health concern, with an estimated 251.4 million people affected worldwide in 2021, and causing at least 10,000 deaths annually.^1^ The disease leads to anemia, growth retardation, abdominal pain, diarrhea, and death, posing a serious threat to both individual health and socio-economic development. Schistosomiasis is categorized into two primary forms: intestinal schistosomiasis and genitourinary schistosomiasis, and is caused by six species of blood flukes: *Schistosoma mansoni* (*S. mansoni*), *Schistosoma haematobium* (*S. haematobium*), *Schistosoma japonicum* (*S. japonicum*), *Schistosoma mekongi* (*S. mekongi)*, and *Schistosoma intercalatum* (*S. intercalatum*).^2^ The *Schistosoma* life cycle alternates between aquatic stages (eggs, miracidium, sporocyst, and cercaria) and sexual reproduction in the definitive host (adult worms). *Oncomelania hupensis* serves as the exclusive intermediate host for *S. japonicum*, while the parasite can mature in at least 46 mammalian definitive hosts, including humans.^1^^,^^3^^,^^4^

In endemic areas, praziquantel (PZQ) is the World Health Organization (WHO)-recommended treatment for all *Schistosoma* species due to its short treatment duration, high efficacy, and low side effects.^1^^,^^5^ Although the molecular mechanism of PZQ remains incompletely understood, the prevailing hypothesis suggests that it disrupts calcium homeostasis in adult worms by affecting voltage-gated Ca^2+^ channels.^6^^,^^7^ Many countries, including China,^8^ the Philippines,^9^ Indonesia,^10^ and Tanzania,^11^ have implemented PZQ in mass drug administration (MDA) programs.^12^ However, decades of extensive use have raised concerns regarding emerging schistosome resistance, supported by laboratory-induced resistance and reduced efficacy after repeated exposure.^13^^,^^14^ A multicountry study assessing therapeutic efficacy through the egg reduction rate (ERR) found that the lower bound of the 95% confidence interval for ERR was 68.4% in *S. japonicum* from the Philippines.^13^ This observation suggests substantial individual variation in drug response. This variability highlights the need for continued monitoring of PZQ efficacy, as reduced responsiveness in a subset of individuals could, if confirmed, signal the early stages of tolerance development. These findings underscore the need to elucidate the genomic basis of potential PZQ action and resistance, as well as to develop new strategies for schistosomiasis control. The long-term and widespread use of PZQ, particularly in Southeast Asia where schistosomiasis prevalence remains high, has likely imposed strong chemotherapeutic selection pressures on *S. japonicum*, providing an evolutionary context for investigating genomic determinants of parasite adaptation.

Among *Schistosoma* species, *S*. *japonicum* is primarily distributed in East and Southeast Asia and comprises four subgroups: the Chinese mainland, Taiwan province, Indonesia, and the Philippines, which is consistent with its geographic distribution.^1^^,^^15^^,^^16^^,^^17^^,^^18^^,^^19^^,^^20^ Based on the mitochondrial DNA, microsatellite, or whole-genome sequencing data, *S. japonicum* was demonstrated to originate from the middle and lower reaches of the Yangtze River (the Lake strains) and then radiated to the mountainous areas of China (the Mountain strain), Japan, and Southeast Asia in the Neolithic period.^18^ The Taiwan population (zoophilic strain) firstly diverged from other populations (zoonotic strain) ∼45000 years ago, consistent with the divergent evolution of intermediate host *O. h. formosana* in Taiwan province and *O.h. tangi* in Fujian province.^21^ Geographic isolation and demographic bottlenecks have resulted in pronounced genetic differentiation between mainland (mountain and lake) and island (Indonesia, Taiwan, and the Philippines) populations, reflected in reduced nucleotide diversity, greater genetic distances, and distinctive physiological traits such as morphology, host susceptibility, prepatent period, and pathology.^22^^,^^23^^,^^24^ In mainland China, the mountain population can infect snails from the lake region, while the lake population cannot infect mountain snails.^25^ Compared with the Chinese lake strain, *S. japonicum* from Taiwan exhibits several unique features, including the inability to infect humans, milder pathology, smaller granulomas, slower development, shorter adult worms, and a longer prepatent period in mice.^22^^,^^23^ These population distinctions offer a valuable natural model for investigating how genetic variation shapes environmental and host adaptation.

Genetic variants can be divided into single-nucleotide variants (SNVs), small insertions or deletions (InDels), and structural variants (SVs), which collectively determine the phenotypic diversity. Our previous study based on SNVs identified several highly differentiated genes among *S. japonicum* subgroups related to host-switching, such as *GATAD2A* and *LCAT* in the Taiwan strain and *Lmln*, *Rab6*, and *VCP* in the mountain population.^22^^,^^26^ While these SNV-based studies have revealed population divergence and host-specific adaptations, the contribution of SVs, a major yet underexplored source of genomic diversity, remains unknown in *S. japonicum*. SVs are genome rearrangements ranging in length from 50 bp to longer than a megabase, including deletion (DEL), insertion (INS), duplication (DUP), inversion (INV), and translocation (BND).^27^ Previous studies have demonstrated that SVs play critical roles in many physiological characteristics and diseases in many species.^28^^,^^29^^,^^30^^,^^31^^,^^32^^,^^33^ For instance, DELs in the cathepsin D gene in *Rhipicephalus microplus* and DUPs in the *CyPJ* gene in *Haemaphysalis longicornis* are likely associated with vector-pathogen adaptation.^34^ SVs also influence the reproductive isolation of the yeast *Schizosaccharomyces pombe*.^35^ However, in parasitic flatworms (Phylum Platyhelminthes), including *Schistosoma* species, the landscape and functional significance of SVs have not yet been systematically characterized.

In this study, we constructed the first genome-wide structural variation (SV) map of *S. japonicum* based on 72 isolates collected from six endemic regions across East and Southeast Asia. By integrating population genetic and functional analyses, we identified highly differentiated SVs and their associated genes linked to PZQ response and reproductive adaptation. These findings provide new insights into how large-scale genomic rearrangements drive parasite evolution and host-parasite interactions, establishing a valuable foundation for future research on schistosome adaptation, drug efficacy, and control strategies.

## Results

### Genomic profiles of structural variations (SVs) in *S. japonicum*

**Figure 1:**
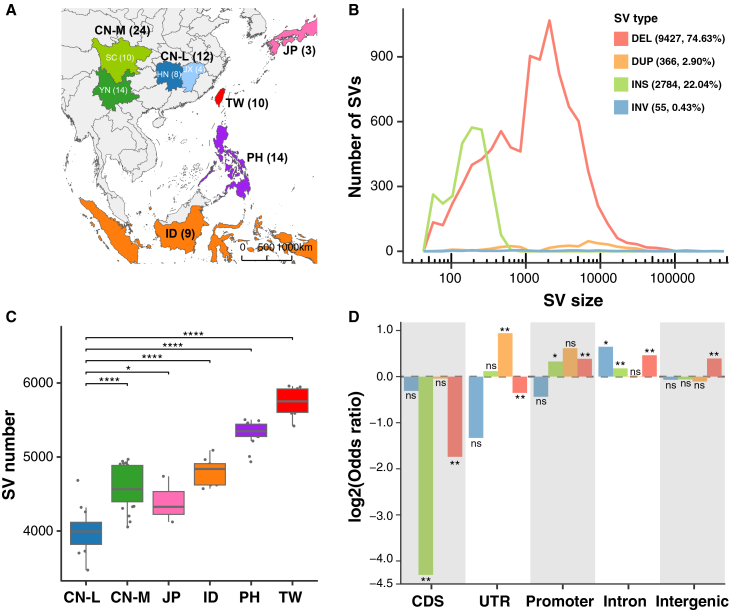
Genomic features of SVs in *S. japonicum* (A) Geographic origins of the 72 *S. japonicum isolates* across East and Southeast Asia. (B) Size distribution of DELs (red color), DUPs (orange color), INSs (green color), and INVs (blue color). (C) Number of SVs identified in each population group. (D) Enrichment analysis of the functional location of SVs across all *S. japonicum* populations. ns: not significant; ∗: *p* value < 0.05; ∗∗: *p* value < 0.01; ∗∗∗: *p* value < 0.001; and ∗∗∗∗: *p* value < 0.0001.

To characterize the genomic landscape of SVs in *S. japonicum*, we analyzed 72 whole-genome sequencing samples (mean read coverage ∼14×, ranging from 7 to 24×) collected from the major endemic regions: 14 from the Philippines (PH), 9 from Indonesia (ID), 3 from Japan (JP) which have limited sample size but provide a broader East Asian context in the SV map, and 46 from China, including 10 from Sichuan (SC) and 14 from Yunnan (YN) (referred as mountain samples, CN-M), 8 from Hunan (HN), 4 from Jiangxi (JX) (referred as low-lying lake samples, CN-L), and 10 from Taiwan (TW) (Figure 1A; Table S1).^22^

SVs were identified using three independent callers: Lumpy,^36^ Manta,^37^ and CNVnator^38^) (see Methods). We generated a high-quality SV map comprising 12,632 SVs across all samples, including 9,427 DEL (74.63%), 366 DUP (2.90%), 2784 INS (22.04%), and 55 INV (0.43%) (Figures 1B, S1A, S1B, and S1D). The numbers of DELs, DUPs, and INSs were strongly correlated with chromosome size (R^2^ > 0.9, *p* < 0.001), suggesting that these SV types accumulate proportionally to genomic content. INVs, however, showed a weaker correlation (R^2^ = 0.67, *p* = 0.013), which may reflect either biological differences in their formation mechanisms or technical limitations of short-reads in detecting INVs. The combined SV length totaled 13.91 Mb, representing 3.55% of the reference genome (392.22 Mb in chromosome-level), including 10.63 Mb of DELs, 2.26 Mb of DUPs, 527 Kb of INSs, and 675 Kb of INVs. SV sizes ranged from 50 bp to 296 kb, with median lengths of 1.4 Kb (DEL), 4.9 Kb (DUP), 178 bp (INS), and 1.2 Kb (INV) (Figure 1B). The number of SVs per sample varied across populations, with median counts of 5,747 (TW), 5,321 (PH), 4,793 (ID), 4,396 (JP), 4,594 (CN-M), and 3,998 (CN-L) (Figures 1C and S2A–S2C). Due to the limited sample size, the Japanese samples were not subjected to population-level comparisons. Then after removing the Japanese samples, these differences were positively associated with genetic distances (*F*_*ST*_) between populations (Figure S2D). Variant allele frequency (VAF) spectra also differed: island populations (TW, PH, ID) exhibited higher proportions of fixed SVs (VAF = 1) than mainland populations (CN-L, CN-M) (Figure S3). Similarly, joint allele frequency spectra (JAFS) between any two populations showed greater dispersion from the diagonal, indicating strong population differentiation (Figure S4). Together, these results demonstrate that geographic isolation has driven extensive SV diversity across *S. japonicum* populations.

We next divided the SVs into five categories based on their positions within the functional elements (see Methods), including coding sequence (CDS), untranslated regions (UTR), promoter, intron, and intergenic regions. We observed that most SVs overlapped with intergenic (51.99%) and intron regions (33.93%), while fewer intersected with CDS (8.15%), UTR (4.01%), and promoter regions (1.92%) (Figure S1C). DELs and INSs were significantly depleted in CDS (functionally important region), while DUPs and INVs were neither enriched nor depleted in that region (Figures 1D and S5), suggesting that purifying selection might be directed against functionally important DELs and INSs, whereas DUPs and INVs might be subjected to some kind of relaxed selection. Interestingly, in UTR regions, DELs were significantly depleted across all populations, and in promoter regions, DELs are significantly enriched in most populations (CN-L, CN-M, ID, and PH), while INSs are significantly enriched in most populations (ID and TW) (Figure S5), implying potential adaptive selection acting on SVs that modulate gene expression and regulatory control.

### Population structure of *S. japonicum* shaped by SVs

**Figure 2:**
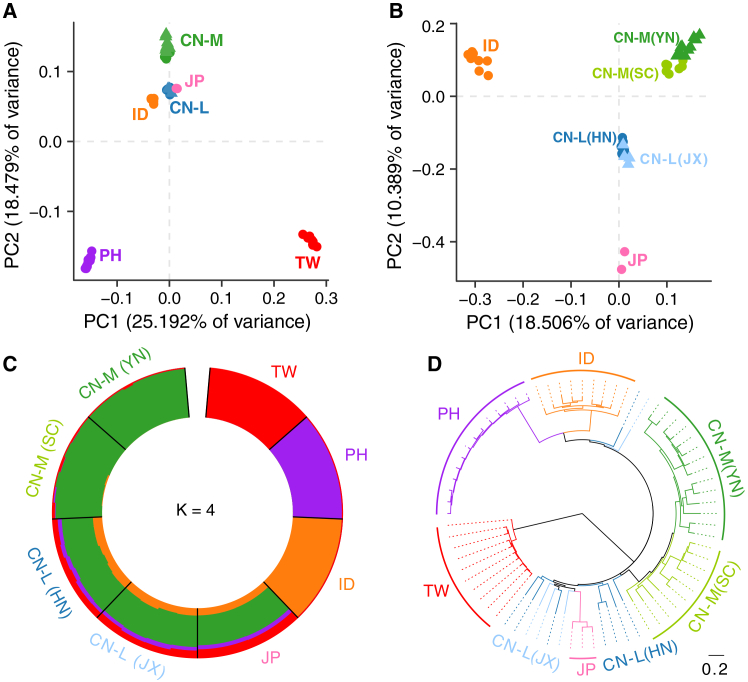
Population structure of *S. japonicum* populations (A) Principal component analysis (PCA) of all *S. japonicum* samples. (B) PCA of samples from CN-L, CN-M, JP, and ID populations showing regional clustering. (C) Ancestry genetic component of all *S. japonicum* samples estimated by ADMIXTURE with the best K = 4. Each color represents one ancestry composition. (D) Maximum-likelihood phylogenetic tree of 72 *S. japonicum* samples inferred from the SVs.

Principal component analysis (PCA) based on SVs revealed clear geographic clustering among *S. japonicum* populations (Figures 2A, 2B, and S6), consistent with the previous SNV-based analyses.^22^ Island populations (TW, PH) were highly divergent from CN-L, CN-M, JP, and ID groups (Figure 2A), consistent with their extremely high genetic differentiation (*F*_*ST*_ *>* 0.3) (Table S2) and SV number difference (Figure 1D). Additionally, the CN-L individuals clustered closely together, whereas the CN-M individuals were more genetically dispersed (Figure 2B). These observations were consistent with the distinct genetic component inferred for these samples using the unsupervised method implemented in the ADMIXTURE software (K = 2 to 10). When K = 4 with the lowest CV error, the TW, ID, PH, and CN-M populations have distinct genetic components, respectively, and those components constructed the genetic component of CN-L (Figures 2C and S7), consistent with the admixture results based on the SNVs.^22^ When K = 5, CN-L and JP share a predominant ancestry component, while each of the other four populations (TW, ID, PH, and CN-M) exhibits a distinct and unique component (Figure S7). This pattern indicates that CN-L and JP are genetically more similar to each other than to other populations, consistent with their placement in a shared clade in the ML phylogenetic tree (Figure 2D). The remaining populations (TW, ID, PH, CN-M) each form genetically distinct groups. When K = 6, distinct genetic components were observed for each population, and CN-M populations showed within-group genetic differentiation, consistent with the genetically dispersed pattern revealed by PCA (Figures 2B and S7). Examination of higher K values (up to K = 10; Figure S7) confirms that this subdivision within CN-M is a stable pattern, persisting across all higher K values, which supports the interpretation of genuine internal genetic structure within the mainland Chinese population rather than a K-specific artifact. These results implied the geographic isolation of TW, ID, and PH populations, and high genetic diversity within CN-L and CN-M populations, which was also supported by the maximum-likelihood (ML) phylogenetic tree (Figure 2D). Overall, SVs' diversity plays a significant role in shaping the diverse population structure in island and mainland *S. japonicum* and potentially facilitates adaptation to varied environments.

### Convergent evolution associated with praziquantel response in island populations

**Figure 3:**
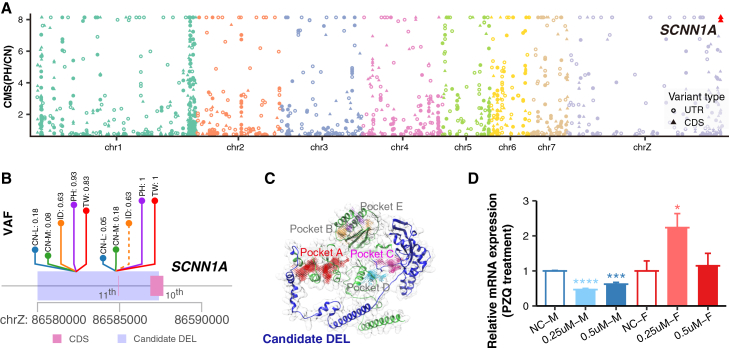
Candidate genes involved in PZQ response in *S. japonicum* (A) Differentiation signals of SVs intersect with the CDS and UTR region between PH and CN. The CMS value represents the sum of the negative log10 transformation of the empirical *p* value of *F*_*ST*_ and dVAF. dVAF: difference in variant allele frequency. (B) The variant allele frequency of DELs in *SCNN1A* genes in CN-L, CN-M, ID, PH, and TW populations. (C) Predicted protein structure including active sites and regions influenced by SVs. Pocket A (red) and Pocket C (purple) are impacted by the DEL (blue), and other pockets (B, D, E) are shown in gray. (D) Relative *SCNN1A* mRNA expression after PZQ treatment in male and female worms. Two-tailed Student’s *t* test was performed for each pairwise comparison. ∗: *p* value < 0.05; ∗∗: *p* value < 0.01; ∗∗∗: *p* value < 0.001; and ∗∗∗∗: *p* value < 0.0001. M: male worm; F: female worm.

To explore the genetic differentiation between island and mainland populations of *S. japonicum*, we calculated genetic differences (*F*_*ST*_) and compared the difference in VAF (dVAF) between each island (9 TWs, 14 PHs, and 8 IDs) and mainland populations (29 CNs) (Figure S8), and focused on the genomic signature in the CDS and UTR region. We identified 229, 231, and 125 significantly highly differentiated genes in TW, PH, and ID, respectively (see Methods), with >20% shared among island groups (Figure S8). Functional enrichment showed overlapping pathways related to calcium-dependent phospholipid binding, lipid binding, phospholipid binding, sodium channel activity, and sodium ion (Na^+^) transmembrane transporter activity (Figure S9). Among these highly differentiated SVs, two DELs overlapped the *SCNN1A* gene encoding the epithelial sodium channel α-subunit (Figure 3; Table S3). These DELs intersected with the CDS region of the *SCNN1A* gene had an extremely high VAF (62–100%) in the island populations but rarely (<18%) in the mainland populations (Figures 3B and S10). Protein structure prediction suggested that these DELs could influence the predicted active pocket of protein, which may affect the transport activity of *SCNN1A* (Figure 3C). PZQ-induced spasmodic contraction of *Schistosoma* was calcium ion (Ca^2+^) dependent but inhibited by high concentrations of extracellular magnesium ion (Mg^2+^). In addition, PZQ also stimulated the influx of Na^+^ but decreased the influx of potassium ion (K^+^).^39^ Previous *in vitro* study showed that PZQ-induced spasmodic contraction of the worm was inhibited when incubated in a medium lacking Na^+^, containing low levels of Ca^2+^, or high concentrations of Mg^2+^, highlighting the important roles of ion channels related to Na^+^, Ca^2+^, and Mg^2+^ in mediating the effects of PZQ in *Schistosoma*.^39^^,^^40^^,^^41^ The *SCNN1A* gene encodes the epithelial Na^+^ channel alpha subunit, a critical regulator of Na^+^ balance and fluid homeostasis across the plasma membrane,^42^ suggesting potential effects on response to PZQ treatment. Therefore, to further explore the roles of *SCNN1A* genes against PZQ treatment, we conducted *in vitro* experiments in *S. japonicum* worms with two concentrations of PZQ treatment (0.25 μM and 0.5 μM) and measured the gene expressions under each treatment group. We observed that after PZQ treatment, the expression of the *SCNN1A* gene significantly increased in female worms but decreased in male worms (Figure 3D), supporting the possible functional role of the *SCNN1A* gene in response to PZQ treatment. Previous research indicated that no evidence for resistance was found in *S. japonicum* from China (mainland populations),^43^ while reduced efficacy (95% CI: 68.4–99.3) was observed in *S. japonicum* within the Philippines (island populations).^13^ Conclusively, the above results suggested that DELs within the predicted active site of *SCNN1A* genes exhibit a convergent evolutionary pattern in island populations and might be the candidate targets linked to PZQ response in *S. japonicum* (Figure 6A).

### SVs contribute to the reproductive development in the Taiwan population

**Figure 4:**
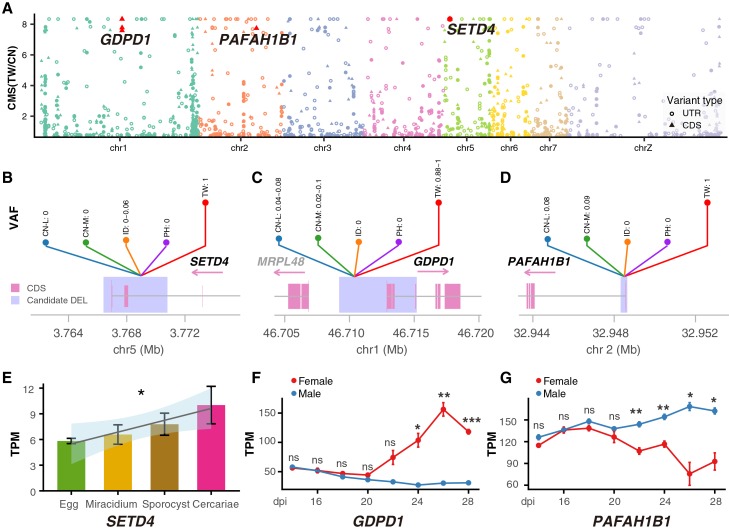
Candidate genes involved in reproduction in the *S. japonicum* Taiwan strain (A) Differential SV signals overlapping with the CDS and UTR region between TW and CN. The CMS value is the sum of the negative log10 transformation of the empirical *p* value of *F*_*ST*_ and dVAF. dVAF: difference in variant allele frequency. (B–D) Variant allele frequencies of DELs in *SETD4, GDPD1,* and *PAFAH1B1* in CN-L, CN-M, ID, PH, and TW populations. (E) Expression of *SETD4* during four aquatic life stages (eggs, miracidium, sporocyst, and cercaria). A linear regression model (log2(TPM+1) ∼ stage, treating stage as an ordinal variable, and Jonckheere–Terpstra test was performed to assess the increasing trend. ∗: *p* value < 0.05. (F and G) Expression of *GDPD1* and *PAFAH1B1* genes at different developmental stages (14–28 days post-infection) in the definitive host. Two-tailed Welch’s t-tests were performed for each pairwise comparison. TPM: transcript per million. ∗: *p* value < 0.05; ∗∗: *p* value < 0.01; ∗∗∗: *p* value < 0.001; and ∗∗∗∗: *p* value < 0.0001.

The *S. japonicum* Taiwan strain was a zoophilic lineage displaying distinct biological phenotypes compared with the Chinese Lake strain, including low pathogenicity with smaller hepatic granulomas, reduced body size of cercaria, and a longer prepatent period, along with reduced egg production in mouse models.^23^^,^^44^ However, the mechanism underlying these unique phenotypes of the *S. japonicum* Taiwan strain remains poorly understood. Among the significantly high-differentiated SVs between TW and CN populations, we detected ten SVs intersecting with three gene regions (*SETD4*, *GDPD1*, and *PAFAH1B1*), all exhibiting extremely high VAF of 100% in the TW population, but nearly absent (0–9%) in other populations (Figures 4A–4D). The DELs overlapped with the 3′UTR and the last CDS region of the *SETD4* gene, which encodes a histone methyltransferase enzyme that could catalyze the trimethylation of histone H3 at lysine 27 (H3K27me3)^45^ (Figure 4B). Previous studies have demonstrated that histone methylation is essential for the life cycle progression of *S. mansoni* and bivalent trimethylation of H3K4 (H3K4me3) and H3K27 (H3K27me3) at the transcript start site (TSS) of genes represents a key epigenetic signature specific to the cercarial stage.^46^ Here, we analyzed the expression profiles of the *SETD4* gene in four stages (eggs, miracidium, sporocyst, and cercaria) in the water phase. Consistent with the bivalently modified histone specific to the cercarial stage, the expression level of SETD4 exhibited a progressive increase during development and presented the highest level in the cercarial stage (Figure 4E). The presence of antagonistic H3K4me3 and H3K27me3 at the TSS of genes has been considered as a mechanism for the temporal and spatial regulation of lineage-controlling gene expression in embryonic stem cells.^47^ Throughout the life cycle of *S. japonicum*, the cercarial stage represents the final phase of asexual multiplication and marks the transition to sexual reproduction. Hence, we hypothesize that DELs located in 3′UTR may regulate the expression of the *SETD4* gene, thereby influencing bivalent histone modification, which plays a potentially important role during the transitory stage from asexual to sexual reproduction (Figure 6B).

**Figure 5:**
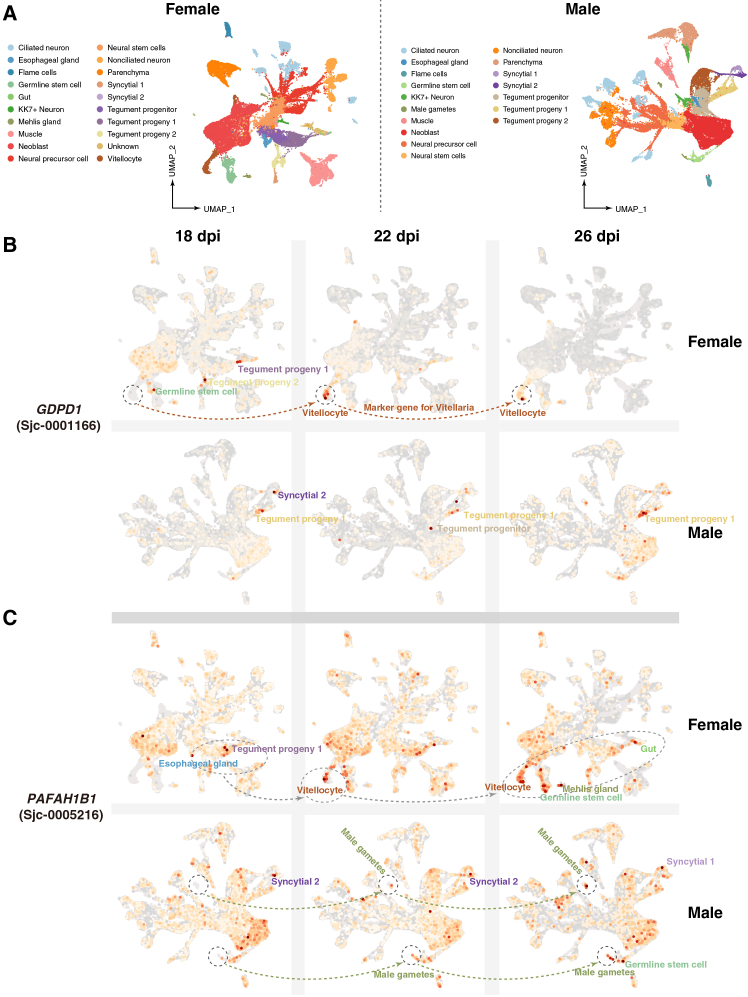
Dynamics expression patterns of *GDPD1* and *PAFAH1B1* genes in distinct cell clusters across developmental stages (A) UMAP visualization of single-cell transcriptomes from female (left) and male (right) samples, classified into 20 and 17 distinct cell types (including one unannotated “unknown” populations). (B) UMAP visualization of the *GDPD1* gene in female (top) and male (bottom) *S. japonicum* at different developmental stages (18, 22, and 26 dpi). (C) UMAP visualization of the *PAFAH1B1* gene in female (top) and male (bottom) *S. japonicum* at different developmental stages (18, 22, and 26 dpi).

**Figure 6:**
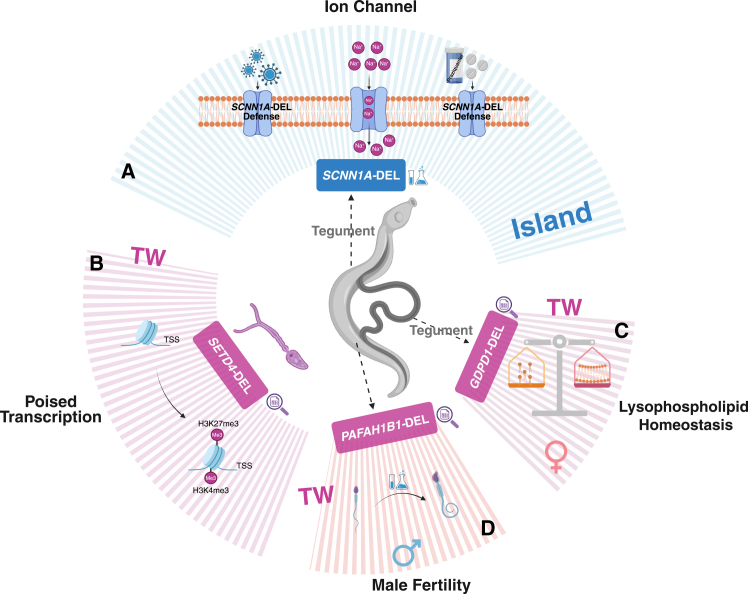
Proposed molecular mechanisms of SVs contributing to drug response and reproductive adaptation in *S. japonicum* (A) Schematic model illustrating how deletions in *SCNN1A* may affect ion transport and contribute to immune defense and response to praziquantel in the island strain (ID, PH, and TW), and model depicting the potential roles of *SETD4* (B), *GDPD1* (C), and *PAFAH1B1* (D) deletions in regulating epigenetic modification, lipid metabolism, and reproductive development in the Taiwan strain. Two distinct visual markers were used to indicate which components of the proposed molecular mechanisms are supported by experimental evidence from this study or previous literature (experimental bottle), and which are inferred based on computational predictions (magnifying glass).

The DELs are located with the first CDS region of the *GDPD1* gene, which encodes an enzyme with lysophospholipase D (lysoPLD) activity toward various lysophospholipids, including lysophosphatidylcholine (lyso-PC), lysophosphatidylethanolamine (lyso-PE), and lysophosphatidic acid (LPA)^48^ (Figure 4C). The tegumental surface membranes of *Schistosoma* are syncytial and covered by two closely apposed lipid bilayers, which are essential for nutrient uptake, host-parasite interaction, and parasite survival.^49^ The membranes are especially enriched in lysophosphatidylserine (lyso-PS) and lyso-PC, and lysophospholipases are responsible for the regulation of phospholipid metabolism, especially the homeostasis of lysophospholipid.^50^ The lipdomic studies in *S. mansoni* have revealed the sex-specific tegumental lipid-composition, and the phospholipids (lyso-PC, PC, and PE) were significantly abundant on the surface of female worms.^51^ In addition, LPA signaling has been demonstrated to affect fertility and reproduction.^52^ Then we analyzed the expression profiles of the *GDPD1* gene from *S. japonicum* at different developmental times (14–28 dpi) in the definitive hosts. A significant increase in the *GDPD1* gene expression was observed in females after the first appearance of mature vitelline cells (20 dpi) (Figure 4F), suggesting a potential role in female reproductive maturation. Furthermore, single-cell RNA-seq data from *S. japonicum* at three key developmental time points (18, 22, and 26 dpi) confirmed that *GDPD1* shows a female-biased developmental switch and was a marker gene for vitellaria. *GDPD1* is expressed in germline stem cells and tegument progeny at 18dpi, but becomes specifically and strongly enriched in vitellocytes after the first appearance of mature vitelline cells (22-26dpi) (Figures 5B and S11A).Vitellocytes are the cells responsible for eggshell synthesis and nutrient provision for developing eggs. Those results provide compelling evidence that *GDPD1* is essential for female reproductive development and egg production. *GDPD1* is also expressed in male tegument-related lineages (Figures 5B and S11B), consistent with its reported lysoPLD-associated functions in membrane biology. Therefore, we hypothesize that DELs located within the first CDS may influence the expression of the *GDPD1* gene, potentially disrupting the lysophospholipid homeostasis and impacting female sexual reproduction and egg production (Figure 6C).

Similarly, DELs were detected in the first coding exon of *PAFAH1B1* (also known as the *LIS1*), which encodes the beta subunit of cytosolic type I platelet-activating factor (PAF) acetylhydrolase (PAF-AH[I]) (Figure 4D). Although the role of PAF in *Schistosoma* is largely unknown, in mammals it is essential for sperm motility, acrosomal function, and male fertility.^53^ *Pafah1b1*-deficient mice exhibited early embryonic lethality.^54^ Expression analysis in bulk RNA-seq of *S. japonicum* revealed sex-specific regulation of *PAFAH1B1* during pairing: Its expression significantly increased in males but decreased in females (Figure 4G). In addition, single-cell RNA-seq data from *S. japonicum* showed that the *PAFAH1B1* gene exhibits broader dynamic expression in both females and males across multiple cells types (e.g., male gametes, vitellocyte, germline stem cells, syncytial cells, tegument progeny, gut, and neoblast) (Figure 5C). In females, *PAFAH1B1* expression was transiently elevated in vitellocytes at 22 dpi, coinciding with vitelline cell maturation, but declined by 26 dpi, when expression became predominantly localized to the gut (Figures 5C and S11B). This transition may reflect a reprioritization of function—from directly supporting vitelline cell activity during the onset of reproduction to enhancing nutrient uptake in the gut to meet the sustained metabolic demands of continuous egg production. However, in males, *PAFAH1B1* expression was low prior to sperm maturation (18 dpi) but became markedly elevated during spermiogenesis (22 and 26 dpi) (Figures 5C and S11B), suggesting a role in late-stage male germ cell development. Hence, we hypothesize that DELs in the *PAFAH1B1* gene could impair male gamete function or pairing efficiency or digestive adaptation for egg production (Figure 6D).

Taken together, these findings suggest that Taiwan-specific DELs within the CDS region of *SETD4*, *GDPD1*, and *PAFAH1B1* genes may underlie the reduced reproductive capacity and low pathogenicity of the *S. japonicum* Taiwan strain by affecting both epigenetic and lipid-regulatory pathways (Figures 6B–6D). Future functional validation of these SVs will be essential to clarify their contribution to host-parasite adaptation and reproductive biology in *S. japonicum*.

## Discussion

This study provided the first whole-genome SV map of *Schistosoma japonicum* across East and Southeast Asia, revealing an overlooked layer of genomic complexity that complements previous single-nucleotide-based studies. By integrating multiple SV callers and population genetic analyses, we uncovered more than 12,000 high-confidence SVs affecting 3.55% of the reference genome, exposing how large-scale genome SV, not just point mutations, shapes parasite diversification, drug response, and host adaptation. This work thus reframes the evolutionary and functional landscape of *S. japonicum* from a SV perspective. To contextualize our findings within the broader schistosome genomics landscape, we compared our SV map with the recently pooled long-read SV catalog for *S. mansoni* (Nair et al. 2025).^55^ That study identified 17,446 SVs in five laboratory populations, covering 6.5% of the *S. mansoni* genome. The higher proportion reported by Nair et al. likely reflects the enhanced sensitivity of long-read technology for detecting complex SVs. This is particularly evident for INVs: Nair et al. detected 311 INVs (1.8% of their SV calls), whereas we identified only 55 (0.43%) in our short-read dataset—a discrepancy consistent with the known limitations of short-read sequencing in resolving complex SVs. Despite these technical differences, important biological commonalities emerge. Both studies underscore the prevalence of DELs and INSs as the major drivers of SV in schistosomes, together accounting for 96.6% of SVs in our study and 97.1% in Nair et al. This convergence suggests that the predominance of these SV types may represent a conserved feature of helminth genome evolution, independent of sequencing technology or species.

The SV-based population structure recapitulated but also refined patterns derived from SNPs,^22^ revealing six regional groups (CN-M, CN-L, TW, JP, PH, ID) that correspond to distinct ecological and epidemiological zones. Notably, the strong correlation between SV divergence and geographic distance highlights the role of historical isolation and local adaptation in driving genome architecture differentiation. Importantly, the enrichment of SVs in regulatory elements, rather than coding regions, suggests that expression modulation rather than protein-coding change may be a dominant evolutionary strategy in *S. japonicum*. Such regulatory plasticity likely enables the parasite to fine-tune developmental and immune-evasive processes across diverse environmental and host contexts. These findings collectively indicate that SVs represent a major, yet previously underappreciated axis of adaptive evolution in parasitic flatworms.

Beyond population divergence, our data reveal molecular signatures of convergent evolution in ion channel and lipid regulatory pathways among island populations, pointing to shared selective pressures from drug exposure and host immunity. We found that over 20% of genes with highly differentiated SVs were shared among island populations. Notably, these island populations are distributed in Southeast Asia, where schistosomiasis remains highly endemic, and PZQ has been extensively deployed through decades of MDA. Such intense and sustained chemotherapeutic pressure likely imposed a common selective landscape, driving parallel genomic adaptation in geographically isolated island strains. Ion channels mediate the transport of charged ions across parasite tegumental membranes and serve essential functions, including parasite survival, immune evasion, and drug extrusion to mediate acquired resistance.^56^^,^^57^^,^^58^^,^^59^^,^^60^^,^^61^ Human populations with long-term residence in tropical regions exhibit adaptive adaptation of proinflammatory responses.^62^ The identification of *SCNN1A* DELs, affecting the epithelial sodium channel α-subunit, as a candidate genomic determinant of PZQ response, introduces a new mechanistic link between ion-channel homeostasis and anthelmintic efficacy. The observed sex-dependent regulation of *SCNN1A* expression under PZQ treatment further suggests sexually dimorphic drug responses, an aspect rarely addressed in schistosome pharmacogenomics. However, several important caveats should be noted. First, we were unable to directly correlate *SCNN1A* DEL frequency with clinical efficacy data (e.g., ERR or cure rate) in the sampled populations, as such data were unavailable for the specific populations. Therefore, any inference regarding a functional role in PZQ susceptibility remains indirect and circumstantial. Second, while the extreme frequency difference between island and mainland populations (62–100% vs. <18%) and its position in the top0.1% of the genome-wide *F*_*ST*_ distribution suggest that selection—rather than drift alone—may be operating at this locus, demographic factors cannot be definitively excluded given the strong bottlenecks experienced by these island populations. Given these limitations, the hypothesis that *SCNN1A* DELs influence drug response should be regarded as a testable proposition requiring direct experimental validation. Future studies should aim to: (1) establish controlled PZQ exposure assays in parasites with defined *SCNN1A* genotypes; and (2) where feasible, conduct clinical sampling protocols that simultaneously collect parasites for genotyping and measure individual treatment outcomes. Until such data are available, the role of *SCNN1A* in PZQ response remains a compelling but unconfirmed hypothesis.

The TW population exhibits zoophilic, low pathogenicity with smaller hepatic granulomas, smaller body size of cercaria, and a longer prepatent period, along with reduced egg production in mouse models (one of the definitive hosts). The Taiwan-specific DELs affecting *SETD4*, *GDPD1*, and *PAFAH1B1*, which define a unique epigenetic and reproductive adaptation. *SETD4* encodes a histone methyltransferase linked to bivalent histone marks (H3K4me3/H3K27me3) crucial for stage transitions, implicating structural modulation of chromatin dynamics in parasite development. *GDPD1*, a lysoPLD, and *PAFAH1B*1, a lipid signaling regulator associated with gametogenesis, together suggest lipid homeostasis as a key determinant of sexual maturation and fecundity. Indeed, single-cell transcriptomics revealed their sex-specific expression patterns aligning with reproductive demands: *GDPD1* becomes specifically enriched in female vitellocytes upon sexual maturation, while *PAFAH1B1* progressively increases in male gametes during spermiogenesis and dynamically shifts from vitellocytes to the gut in females. This supports a model where lipid metabolism is spatiotemporally regulated to meet the distinct biosynthetic and energetic needs of male and female reproduction. These genes, disrupted by population-specific SVs, may underlie the attenuated pathogenicity and restricted host range of the Taiwan strain.

Taken together, this work establishes SV as an important driver of genome evolution in *S. japonicum*, providing a new conceptual framework for understanding parasite adaptation at multiple biological scales: from convergent evolution, sexual differentiation, to drug response. The identified SVs in *SCNN1A, SETD4*, *GDPD1*, and *PAFAH1B1* represent candidate molecular levers linking environmental pressure, epigenetic regulation, and pharmacological outcomes. Beyond providing a high-resolution genomic resource, our findings lay the groundwork for SV-based molecular surveillance of PZQ efficacy and rational discovery of next-generation anthelmintics targeting ion channel and lipid metabolic pathways, opening translational opportunities for precision control of schistosomiasis, For example, the population-diagnostic SVs identified here—particularly those fixed or at high frequency in specific endemic regions—can be converted into low-cost PCR-based assays by targeting their unique breakpoint sequences. This approach enables rapid genotyping of individual parasites or pooled field samples without the need for whole-genome sequencing, making it cost-effective and scalable for resource-limited settings where schistosomiasis is endemic. Furthermore, these markers can be integrated into existing surveillance frameworks. In China, for instance, national schistosomiasis control programs already employ sentinel site monitoring to track infection rates and snail distribution.^63^ Incorporating SV-based assays into these sentinel sites would allow tracking of parasite population dynamics—such as the spread of specific lineages, introgression between previously isolated populations, or the emergence of variants potentially associated with drug tolerance—alongside conventional epidemiological indicators.

### Limitations of the study

Although our study provides the first landscape of SVs in *S. japonicum* across major endemic regions, there might still be under-representation in some local areas. Firstly, the limited sample size for the Japanese population (*n* = 3) may not capture the full allelic spectrum of SVs in Japan. The observations regarding the JP should be treated as descriptive and preliminary. However, schistosomiasis japonica was officially declared eradicated in Japan by 1996,^2^^,^^64^ and the last reported human infection occurred decades earlier (1977).^2^^,^^65^ No contemporary field isolates are available for further collection. Therefore, all conclusions involving the Japanese population should be interpreted with appropriate caution, and the data presented here constitute a rare, non-renewable genetic resource. This is particularly valuable for SV research, as such irreplaceable isolates help inform the population-scale SV landscape and provide a historical baseline for interpreting structural diversity across endemic regions.

Besides, all SV in this study were detected relative to the mainland (Anhui) Chinese reference. Detection sensitivity may be reduced for highly divergent lineages (e.g., the Taiwan isolate), potentially leading to the underrepresentation of lineage-specific SVs in regions with greater sequence divergence. The construction of a pan-genome in the future will be crucial for mitigating such bias and enabling more comprehensive and equitable SV discovery. Additionally, the use of the short-read sequencing technology has limitations in detecting SVs in complex genomic regions, such as segmental DUPs, highly repetitive sequences, or regions with extreme GC content. This technical limitation may partially explain the notably low number of INVs observed in our dataset, which could be an underrepresentation of the true biological landscape. Future studies employing long-read sequencing technologies (e.g., PacBio HiFi, Oxford Nanopore) will be essential to more comprehensively resolve structural complexity and breakpoint architecture across *S. japonicum* populations; however, routine individual-level long-read sequencing in schistosomes remains constrained by the limited yield of high-molecular-weight DNA from single worms and will require further methodological advances in low-input long-read library preparation.

While we have identified many potentially valuable SVs, the detailed effect of SVs on gene function remains unclear and requires experimental validation (e.g., stage-specific expression profiling following gene perturbation). This study primarily focused on SVs located within the CDS and UTR regions of the gene, while SVs in the regulatory region might contribute to the adaptation to diverse environments and deserve further investigation. Furthermore, additional genes having potentially biological or evolutionary importance should be investigated in further study.

## Resource availability

### Lead contact

Further information and requests for resources regarding this work should be directed to and will be fulfilled by the lead contact, Yan Lu (lueyan@fudan.edu.cn).

### Materials availability

This work did not generate any unique reagents.

### Data and code availability

The genome-wide SV dataset of *Schistosoma japonicum* generated in this study has been deposited in Mendeley Data : https://doi.org/10.17632/9z2wsd63tp.2. The whole-genome Raw sequencing data of 72 *S. japonicum* are available from NCBI database (BioProject: PRJNA789681). The reference genome ASM2146165v1 (GenBank: GCA_021461655.1) is available from NCBI database (BioProject: PRJNA739049). The transcriptome data used in this study were obtained from NCBI database (BioProject: PRJNA719283 and BioProject: PRJNA343582). This paper does not report original code. Any additional information required to reanalyze the data reported in this work paper is available from the lead contact upon request.

## Acknowledgments

This work was supported by the National Key Research and Development Program of China (no. 2023YFC2605400), the National Natural Science Foundation of China (NSFC) grants (32470649, 32288101), the Shanghai Science and Technology Commission Program (QNKJ2024023, 25JS2810100, 23JS1410100, 24JS2840300), Fundamental and Interdisciplinary Disciplines Breakthrough Plan of the Ministry of Education of China (JYB2025XDXM508), the Fund of Fudan University and Cao’ejiang Basic Research (24FCB08) and the Office of Global Partnerships (Key Projects Development Fund). The computational work in this study was supported by the CFFF Computing Platform and the Human Phenome Data Center of Fudan University. We thank the staff at the National Institute of Parasitic Diseases, Chinese Center for Disease Control and Prevention (NIPD, China CDC) for providing the cercaria of *S. japonicum*.

## Author contributions

Y.L. conceived and designed the study and supervised the project. Q.L. and K.Y. analyzed the data. Q.L. drafted the manuscript. W.Z. performed the experiment. W.H. provided the *Schistosoma japonicum* experimental platform and assisted with data curation. Y.L. and S.X. revised the manuscript. All authors discussed the results and implications and commented on the manuscript.

## Declaration of interests

The authors declare no competing interests.

## STAR★Methods

### Key resources table

**Table:** 

REAGENT or RESOURCE	SOURCE	IDENTIFIER
**Deposited data**		

*Schistosoma japonicum* genome ASM2146165v1	Luo et al.^21^	GenBank: GCA_021461655.1; BioProject: PRJNA739049
72 resequenced *S. japonicum* genomes	Luo et al.^21^	BioProject: PRJNA789681
Adult *S. japonicum* transcriptome	Wang et al.^64^	BioProject: PRJNA343582
Larval *S. japonicum* transcriptome	Cheng et al.^65^	BioProject: PRJNA719283
SV data of 72 *S. japonicum*	This paper	Mendelay Data: https://doi.org/10.17632/9z2wsd63tp.2

**Software and algorithms**		

BWA (v0.7.17)	Li and Durbin^66^	RRID: SCR_010910; https://bio-bwa.sourceforge.net/
Picard (v2.20.2)	Broad Institute	RRID: SCR_006525; http://broadinstitute.github.io/picard/
VCFtools (v 0.1.17)	Danecek et al.^67^	RRID: SCR_001235; https://github.com/vcftools/vcftools/releases
BEDTools (v2.30.0)	Quinlan and Hall^68^	RRID: SCR_006646; https://bedtools.readthedocs.io/en/latest/
PLINK (v1.9)	Chang et al.^69^	RRID: SCR_001757; https://www.cog-genomics.org/plink/
IQ-TREE (v1.6.12)	L.-T. et al.^70^	RRID: SCR_017254; http://www.iqtree.org/
ADMIXTURE (v1.3.0)	D.H. et al.^71^	RRID: SCR_001263; http://dalexander.github.io/admixture/
AncestryPainterV2	Chen et al.^72^	https://github.com/Shuhua-Group/AncestryPainterV2
KING (v2.3.0)	Manichaikul et al.^73^	RRID: SCR_009251; https://www.kingrelatedness.com/
clusterProfiler (v4.2.2)	Wu et al.^74^	RRID: SCR_016884; https://github.com/YuLab-SMU/clusterProfiler
AlphaFold2	Jumper et al.^75^	RRID: SCR_025454; https://colab.research.google.com/github/sokrypton/ColabFold/blob/main/AlphaFold2.ipynb#scrollTo=11l8k--10q0C
POCASA (v1.1)	Yu et al.^76^	https://g6altair.sci.hokudai.ac.jp/g6/service/pocasa/
UCSF Chimera (v 1.16)	Pettersen et al.^77^	RRID: SCR_004097; https://www.cgl.ucsf.edu/chimera/
R	R Core Team	RRID: SCR_001905; https://www.r-project.org/
Manta (v1.6.0)	Chen, X. et al.^36^	RRID: SCR_022997; https://github.com/Illumina/manta
Lumpy (v0.2.13)	Layer et al.^35^	RRID: SCR_003253; https://github.com/brentp/smoove
CNVnator (v0.3.3)	Abyzovet al.^37^	RRID: SCR_010821; https://github.com/abyzovlab/CNVnator
Graphtyper2 (v2.7.4)	Eggertsson et al.^78^	https://github.com/DecodeGenetics/graphtyper
svimmer (v0.1)	Eggertsson et al.^78^	https://github.com/DecodeGenetics/svimmer
Bcftools (v 1.12)	Danecek et al.^79^	RRID: SCR_005227; https://github.com/samtools/bcftools
Trim_galore (v0.6.7)	NA	RRID: SCR_011847; https://github.com/FelixKrueger/TrimGalore
HISAT2 (v2.2.1)	Kim et al.^80^	RRID: SCR_015530; https://github.com/DaehwanKimLab/hisat2
HTSeq (v2.0.2)	Anders et al.^81^	RRID: SCR_005514; https://github.com/htseq/htseq
EdgeR package (v3.38.2)	Robinson et al.^82^	RRID: SCR_012802; https://bioconductor.org/packages/release/bioc/html/edgeR.html
BISER (v1.4)	Iseric et al.^83^	https://github.com/0xTCG/biser

### Experimental model and study participant details

C57BL/6 mice were purchased from SPF 717 (Beijing) Biotechnology Co., Ltd. C57BL/6 mice was infected with *S. japonicum* cercariae, and adult worms were collected at 24 days post-infection (dpi) which was the time point of sexual maturity.^66^ Adult *S. japonicum* were carefully isolated from the portal and mesenteric veins under a stereomicroscope and extensively washed with sterile phosphate-buffered saline (PBS) to remove any adhering host tissue or blood cells. Worms were sex-separated for downstream analyses. For drug treatment, male and female worms were exposed to 0.25 μM or 0.5 μM praziquantel for 24 h, with four biological replicates per condition.

### Method details

#### Ethics statement

All experiments involving animals were carried out in accordance with the guidelines for the Care and Use of Laboratory Animals of the Ministry of Science and Technology of the People’s Republic of China and approved by the Animal Care and Use Committee of Fudan University (Fudan IACUC 201802158S).

#### Data collection and alignment

The re-sequenced data of 72 *S. japonicum* were downloaded from the NCBI (SRA: PRJNA789681).^22^ Reads of 72 *S. japonicum* samples were aligned to the reference genome (ASM2146165v1) using BWA-MEM (v0.7.17)^67^ and Picard software (v2.20.2) was used to mark duplicates of the bam files. We directly used The SNP dataset previously generated and published by Luo et al. 2022.^22^ The relatedness was calculated by KING (v2.2.8) software^68^ using autosome SNP data, and 62 unrelated samples were retained for some analysis (Table S1).

#### SV discovery and population-scale genotyping

Per-sample SVs detection was carried out on 72 *S. japonicum* samples using Lumpy (v0.2.13),^36^ Manta (v1.6.0),^37^ and CNVnator (v0.4.1)^38^ with default parameters. Referring to the recommentation and systematic evaluation,^69^^,^^70^ the following criteria were subsequently applied to filter the SVs detected by different software, i.e., for Manta, DELs/INVs/INSs with “FILTER = PASS” and DUPs with “FILTER = PASS” & INFO/SVLEN<1000 bp; for Lumpy, DEL with INFO/SU > 3, DUP with INFO/SU > 10 & INFO/SVLEN<1000 bp, and INV with INFO/SU > 10; for CNVnator, SVs with natorQ0<0.5 & INFO/SVLEN>1000 bp. Referring to the protocol for SV calling,^71^ for each sample, SVs discovered by Manta, Lumpy, and CNVnator were merged using svimmer (v0.1, https://github.com/DecodeGenetics/svimmer)^70^ with default parameters. Any SV (except for insertions) with a length larger than 2 Mb or smaller than 50bp was filtered out. Then, all SVs detected in all samples were merged again by svimmer with the same filters. Lastly, population-scale genotyping was performed using Graphtyper2 (v2.7.7)^70^ and only results based on the aggregate model were retained. For inversions, results with the breakpoint model were retained if no aggregate model was provided for it. Subsequently, a series of criteria was conducted for a high-confidence SV filter. Firstly, we applied the following filters using vcffilter from vcflib (https://github.com/vcflib/vcflib): vcffilter -f “(SVTYPE = BND & QD > 20 & ( ABHet >0.30 | ABHet <0) ) | (SVTYPE = DEL & QD > 12 & ( ABHet >0.30 | ABHet <0) ) | (SVTYPE = DUP & QD > 5) | (SVTYPE = INS & ( ABHet >0.25 | ABHet <0) & MaxAAS >4) | (SVTYPE = INV & ( ABHet >0.25 | ABHet <0) & MaxAAS >4)ˮ.^70^ Then, sites with over 30% overlap with simple repeats and DNA satellite or over 5% overlap with segment duplication region identified by BISER (v1.4)^72^ were filtered out. Further, low-quality SV genotype calls with GQ < 12 were also filtered out, and then sites with a missing rate >20% were removed.

#### Gene feature of SVs

The general feature format (GFF) file for the reference genome (ASM2146165v1) was used to annotate detected SVs. We defined the precedence of functional elements owing to one SV possibly intersecting with multiple features: coding sequence (CDS) > untranslated region (UTR) > promoter > intron > intergenic regions. The promoter region was defined as the 1 kb upstream region of the transcription start site (TSS) of a gene. The intersection of SVs was detected by using BEDTools (v2.30.0).^73^

To explore the enrichment of SVs in each genomic feature (e.g., CDS or UTR), we first generated 1000 random shuffled SV sets within the accessible (non-masked) portion of the genome, excluding simple repeats, DNA satellite, and segment duplication regions, as a null distribution using the shuffle function in BEDTools with the -excl options to constrain shuffling to accessible regions,^73^ including the same number of each type of SVs, the same chromosome, and the same size as the observed SV set. The enrichment value was then defined as the observed SV count in a certain category divided by the mean SV counts in the null distribution, followed by log2 transformation. Positive (negative) value indicated that SVs were enriched (depleted) in a certain category.

#### Population stratification and differentiation analysis

Principal component analysis (PCA) was used to reveal the population structure of *S. japonicum based* on SV. The SVs with a high linkage disequilibrium were filtered out by PLINK with --indep-pairwise 50 5 0.5, and then the PCA was conducted by PLINK (v1.9).^74^ The phased SV data was generated by BEAGLE (v27Feb25.75f) along with SNV data. The phylogenetic tree was constructed by IQ-TREE (v3.0.1)^75^ with parameters “-m MFP -bb 1000ˮand then visualized with FigTree (https://github.com/rambaut/figtree). The ancestry components were estimated by ADMIXTURE^68^ and then visualized by AncestryPainterV2.^76^

The *F*_*ST*_ statistic between populations was calculated by using PLINK (v1.9)^74^ to detect the SVs with high differentiation that underwent natural selection. In addition, the difference in variant allele frequency (dVAF) between populations was calculated, and 1000 random permutated SV sets were also generated to construct the empirical distribution of the *F*_*ST*_ statistic. SV with an empirical *p* value smaller than 0.001 and dVAF>0 was considered as a candidate SV undergoing natural selection. We performed functional GO enrichment of genes using the clusterProfiler^77^ package in R. The threshold was set as P-value ≤ 0.05.

#### Functional characterization of genes

To explore the impact of praziquantel on the expression of candidate genes, we conducted four biological replicates for each treatment group (0.25 μM and 0.5 μM) in male and female *S. japonicum*, along with four untreated controls. After praziquantel treatment, gene expression was monitored by qRT-PCR, and the statistical analysis of gene expression was performed in GraphPad Prism software using the two-tailed Welch’s *t* test.

To investigate the functions of candidate genes across the life cycle, we downloaded RNA-seq data of *S. japonicum* in the four early developmental stages (eggs, miracidium, sporocyst, and cercaria, NCBI: PRJNA719283)^78^ and data of male and female worms across the sexual maturation process in the final host (14–28 days post infection (dpi), NCBI: PRJNA343582).^66^ We use Trim_galore (version 0.6.7) with parameters “paired --length 70 --quality 20ˮ to perform quality control on the raw data. Then the high-quality reads were mapped to the reference genome of *S. japonicum* (ASM2146165v1) by HISAT2 (version 2.2.1)^79^ and then converted into the gene-level counting matrices by HTSeq with parameters “-s no -r pos --mode = union --typeexon–idattrgene_idˮ.^80^ Finally, edgeR (version 4.6.3)^81^ was used for data normalization and expression estimation with the transcript per million (TPM) method. Trend analysis across ordered developmental stages (eggs, miracidium, sporocyst, and cercaria) was performed using linear regression on log2(TPM+1) values, with stage treated as an ordinal variable. To confirm the monotonic trend, the non-parametric Jonckheere-Terpstra test was also applied. Statistical significance was set at *p* < 0.05. Two-tailed Welch’s t-tests were performed for comparison between males and females at each dpi (14-28dpi). A *p* value <0.05 was considered statistically significant.

To investigate the cell-type specific expression of *GDPD1* and *PAFAH1B1*, we utilized single-cell RNA-seq data from *S. japonicum* at 18, 22, and 26 dpi. These data were originally generated and published by our revious study^82^ and are available at NCBI database (PRJNA1244463 and GSE293642).^82^ Processed data, including cell type annotations and gene expression visualizations (e.g., UMAP plots, bubble plots), were used directly in this study. All cell type classifications (e.g., tegument progeny, vitellocytes, male gametes) follow the annotations established in the original publication.^82^ No additional computational analysis of the raw data was performed.

To investigate the impact of candidate SVs on three-dimensional protein structures of candidate genes, we firstly used the AlphaFold2 webserver with default parameters to predict the structure of the target proteins (https://colab.research.google.com/github/sokrypton/ColabFold/blob/main/AlphaFold2.ipynb#scrollTo=kOblAo-xetgx).^83^ Then we used the POCASA webserver with default parameters to predict the active pockets of the target protein (https://g6altair.sci.hokudai.ac.jp/g6/service/pocasa/).^84^ Lastly, protein structures were visualized by UCSF Chimera.^85^

### Quantification and statistical analysis

All statistical analysis was performed using R software unless stated otherwise,. All tests were two-tailed, with statistical significance set at a *p*-value below 0.05.

## Supplemental information

Document S1. Figures S1–S11

Table S1. Sample information, related to Figure 1

Table S2. Mean Fst between each population, related to Figure 2

Table S3. The candidate selective SVs, related to Figures 3 and 4
