# Supplementary material for: Structural variation drives praziquantel response and host adaptation in Schistosoma japonicum
Source: iScience. 2026 May 27;29(6):116118. doi: 10.1016/j.isci.2026.116118 (PMC13235503; doi:10.1016/j.isci.2026.116118)
Supplement: Document S1. Figures S1–S11 [file mmc1.pdf]

iScience, Volume *29*

Supplemental information

Structural variation drives praziquantel response and host adaptation in *Schistosoma japonicum*

Qi Liu, Ke Yang, Wei Zhang, Shuhua Xu, Wei Hu, and Yan Lu

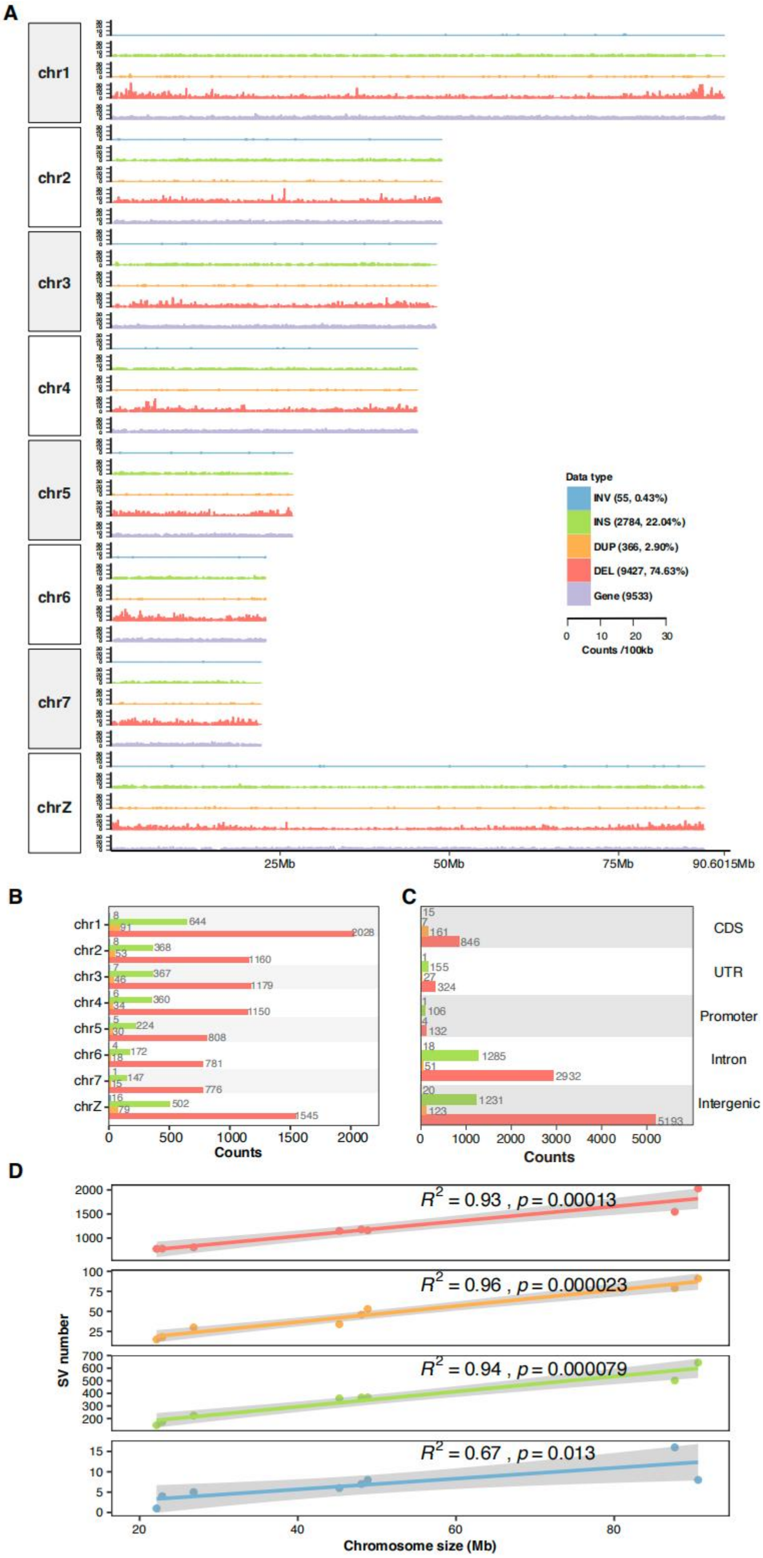

Figure S1. SVs feature of *S. japonicum*, Related to Figure 1.

(A) Genomic locations of SVs for *S. japonicum*. **(B)** SV number in each chromosome in *S. japonicum*. **(C)** Functional overview of SV in S. japonicum. **(D)** The relationship between chromosome size and SV number.

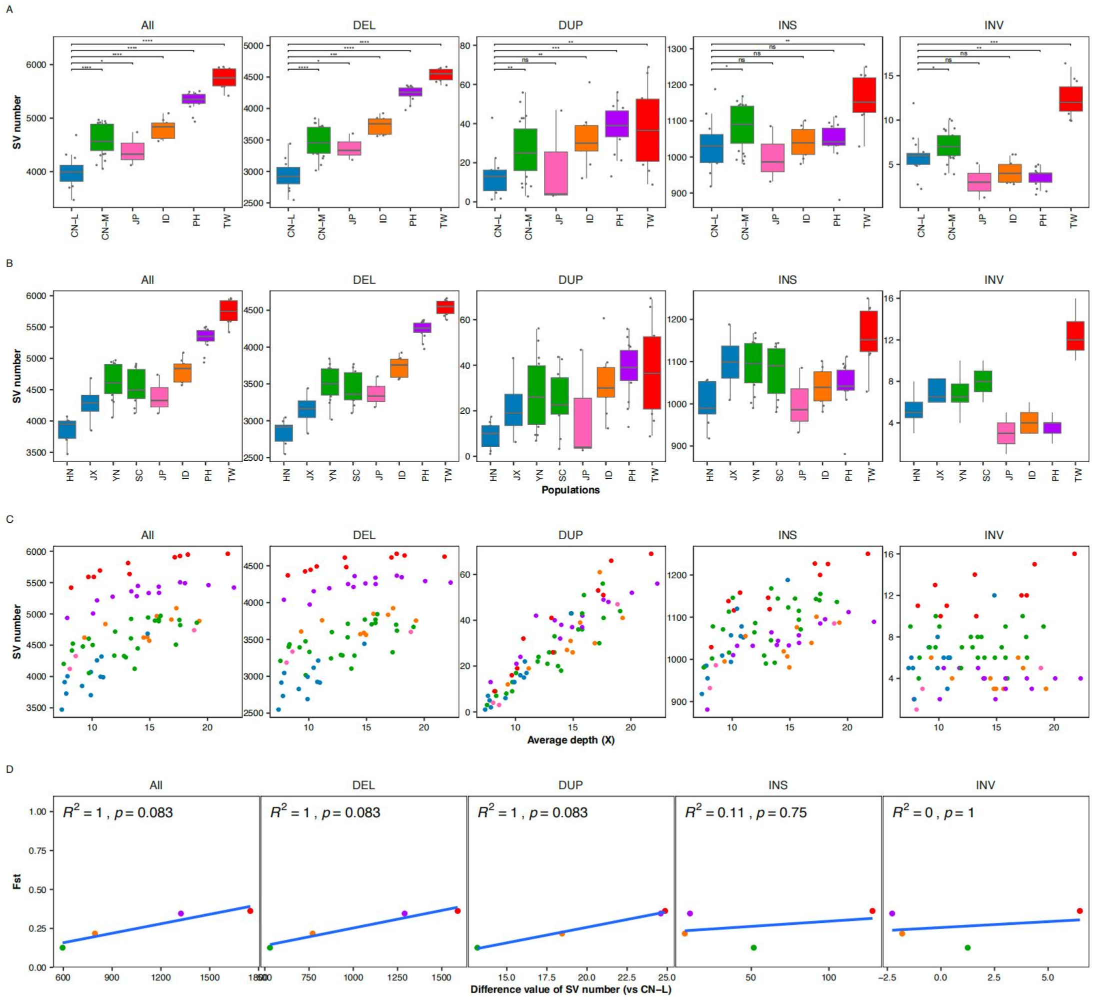

Figure S2. SVs number in different populations of *S. japonicum*, **Related to Figure 1.**

(A, B) The number of different types of SVs across different populations (CN-L, CN-M, JP, ID, PH, and TW) of *S. japonicum*. **(C)** The number of different types of SVs in different sequencing depths. **(D)** The relationship between the difference in SV number with CN-L and the genetic difference (F_{ST}).

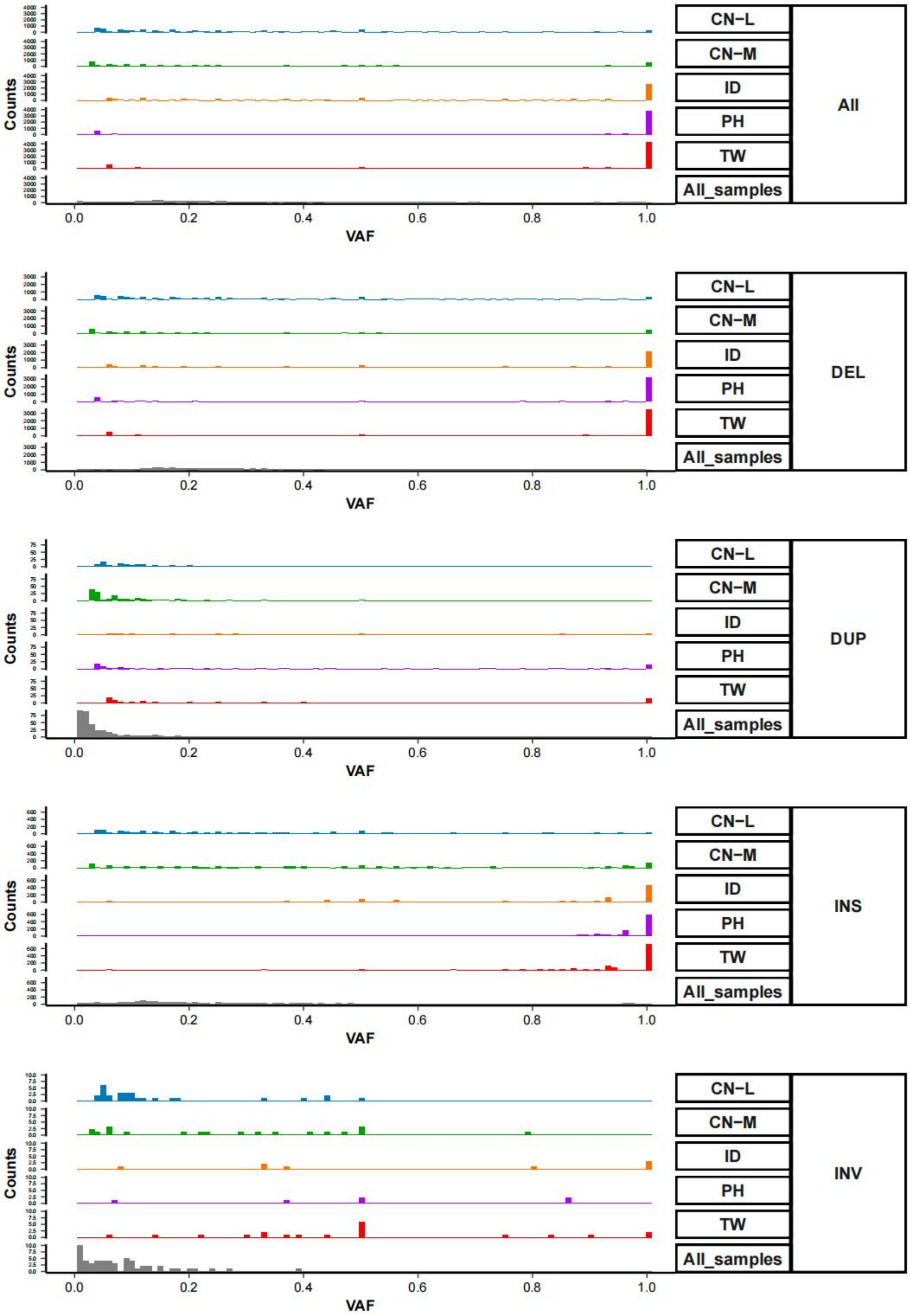

Figure S3. The variant allele frequency of various SV types in different populations of *S. japonicum*, Related to Figure 1.

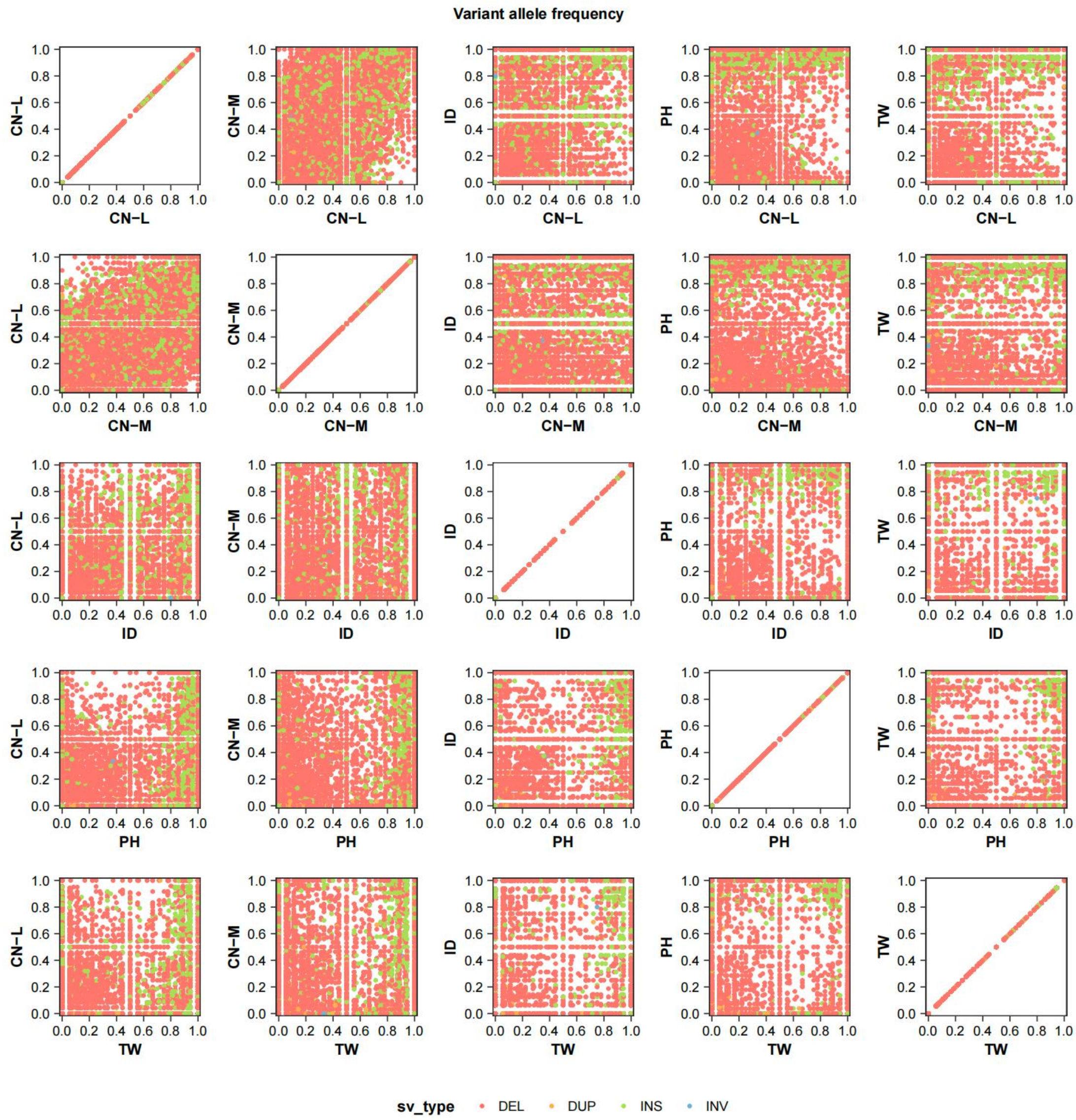

Figure S4. The joint allele frequency spectrum (JAFS) between any two populations in *S. japonicum*, Related to Figure 1.

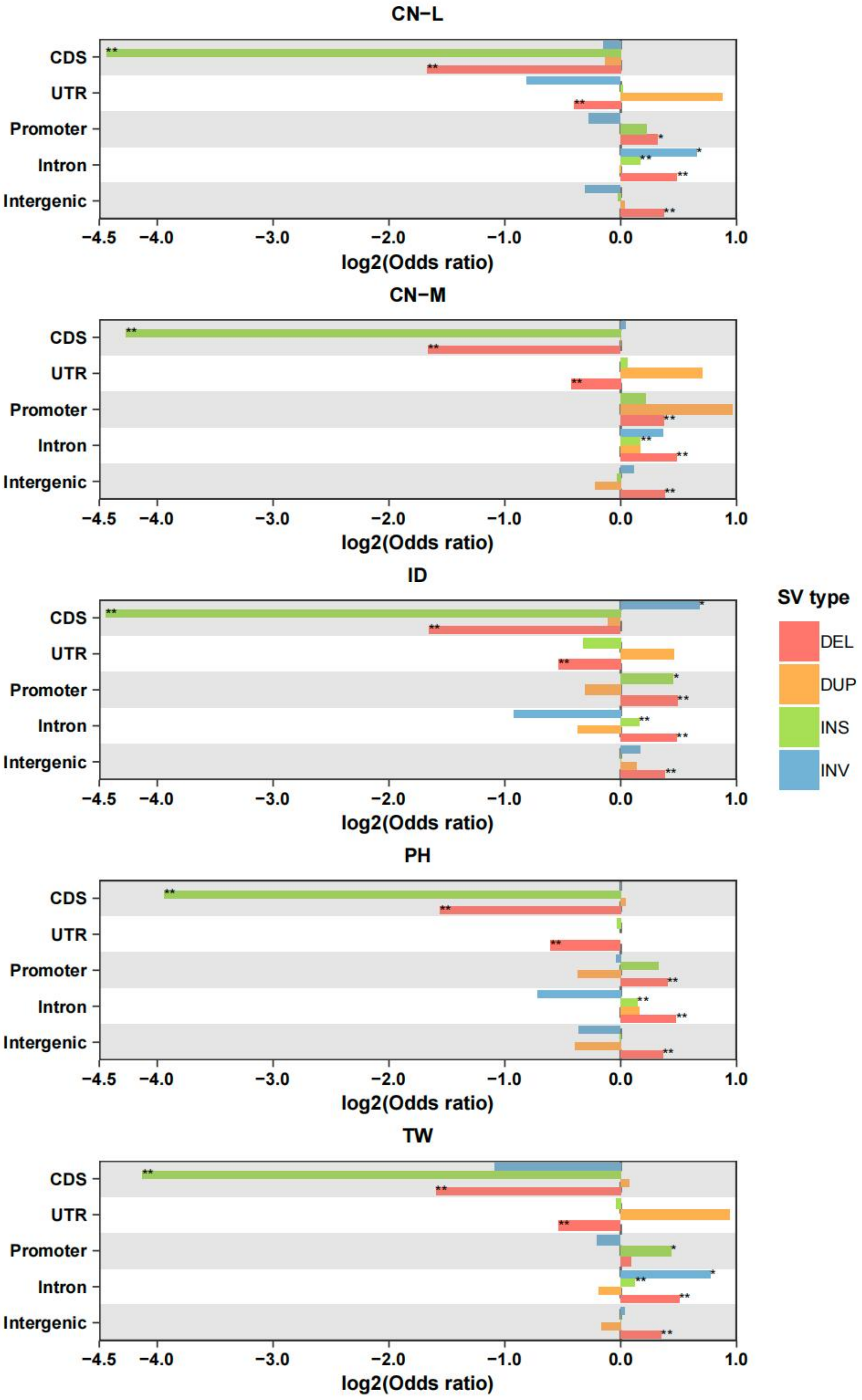

Figure S5. The Enrichment analysis of the functional location of SVs in different populations in *S. japonicum*, Related to Figure 1.

ns: not significant; *: p value <0.05; **: p value<0.01.

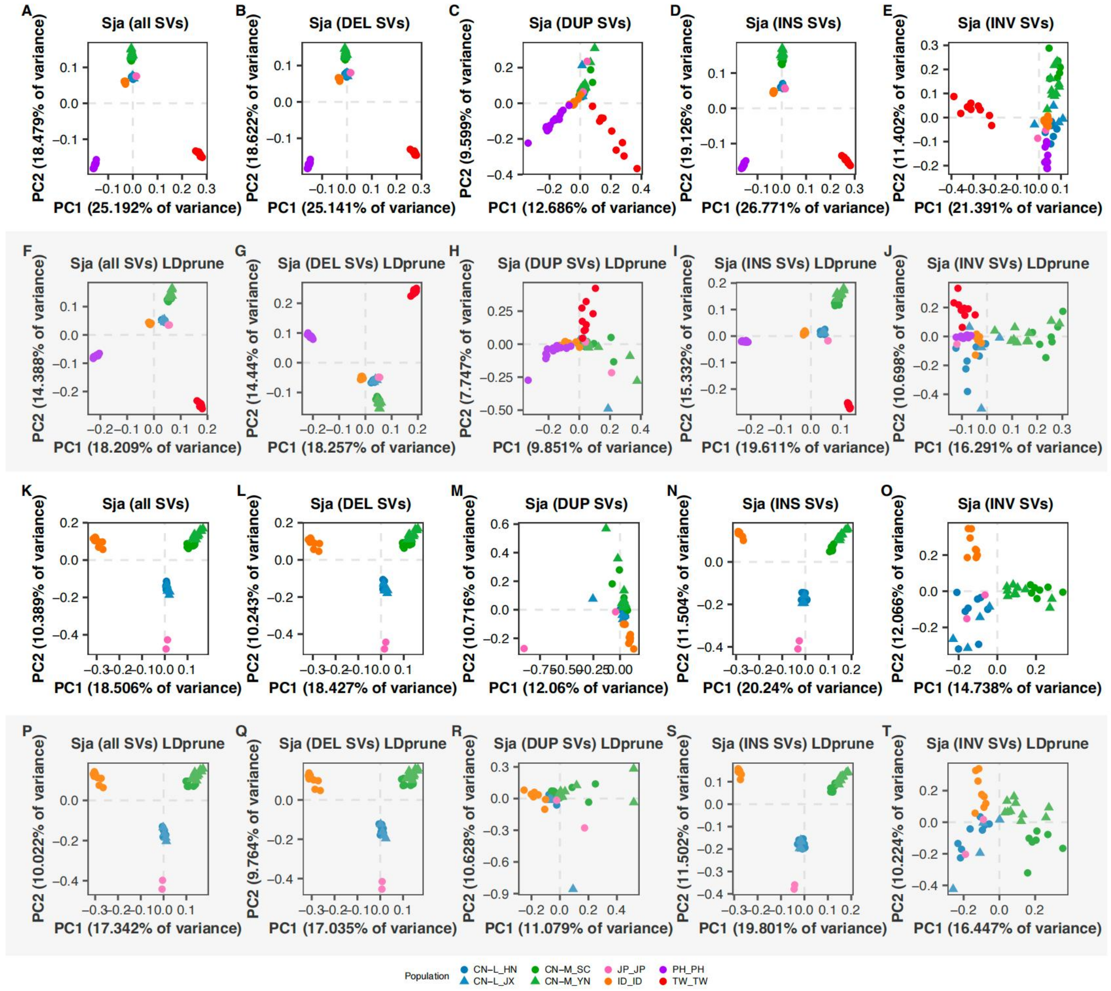

Figure S6. Population structure of *S. japonicum* populations, Related to Figure 2.

(A-J) Principal-component analysis (PCA) result of all the *S. japonicum* samples. **(K-T)** PCA result of the *S. japonicum* samples from CN-L, CN-M, JP, and ID.

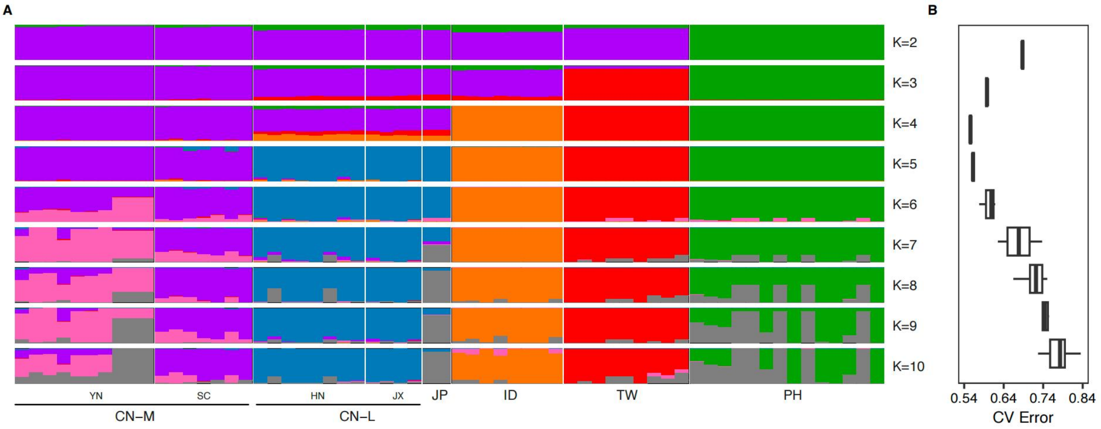

Figure S7. Admixture results of *S. japonicum* populations, Related to Figure 2.

(A) Ancestry genetic component of all S. japonicum samples estimated by ADMIXTURE with the K from 2 to 10. Each color represents one ancestry composition. **(B)** The CV error corresponding to each K value.

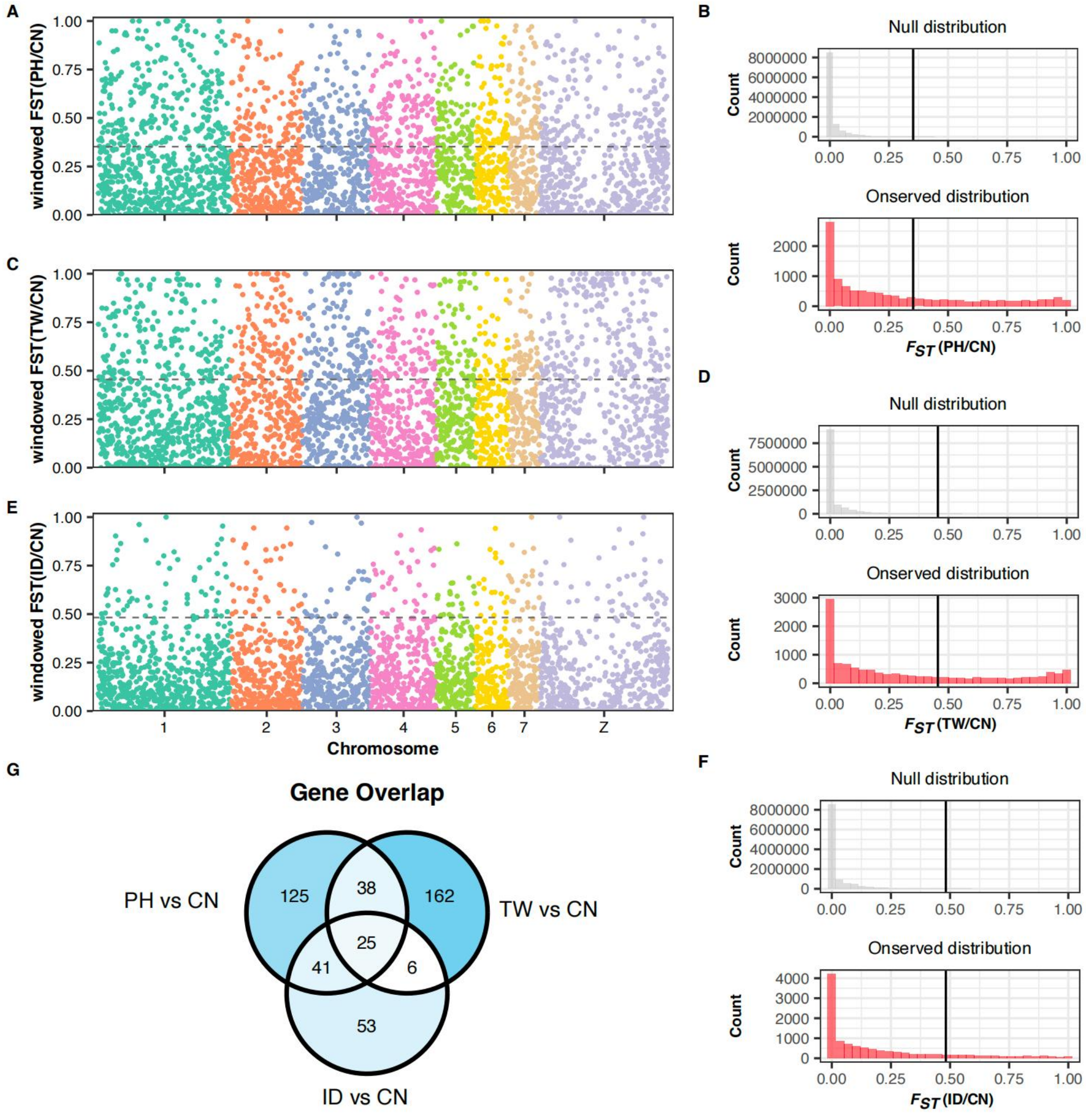

Figure S8. The result of natural selection analysis in *S. japonicum*, Related to Figure 3 and 4. **(A)** The windowed F_{ST} statistics between PH and CN populations. Window size is 100kb and step size is 50kb. **(B)** The null distribution and observed distribution of FST statistics between PH and CN populations. **(C)** The windowed FST statistics between the TW and CN populations. Window size is 100kb and step size is 50kb. **(D)** The null distribution and observed distribution of FST statistics between the TW and CN populations. **(E)** The windowed FST statistics between ID and CN populations. Window size is 100kb and step size is 50kb. **(F)** The null distribution and observed distribution of FST statistics between ID and CN populations. **(G)** The Venn diagram of significant selective genes between each comparison.

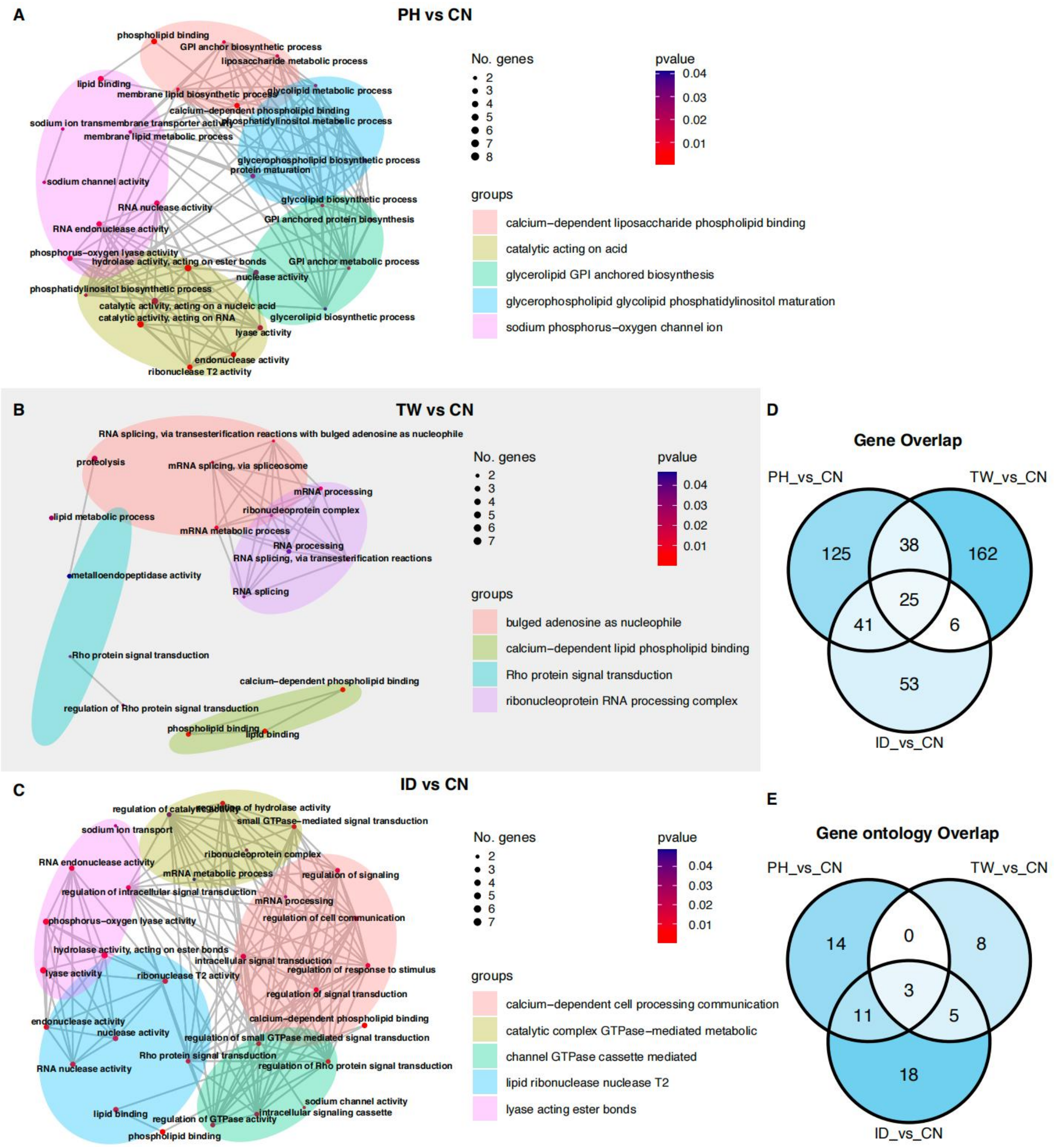

Figure S9. The functional enrichment analysis in *S. japonicum*, Related to Figure 3 and 4.
(A-C) The function enrichment analysis for highly differentiated genes between PH and CN, ID and CN, and TW and CN populations. **(D)** The Venn diagram of significant selective genes between each comparison. **(E)** The Venn diagram of gene ontology between each comparison.

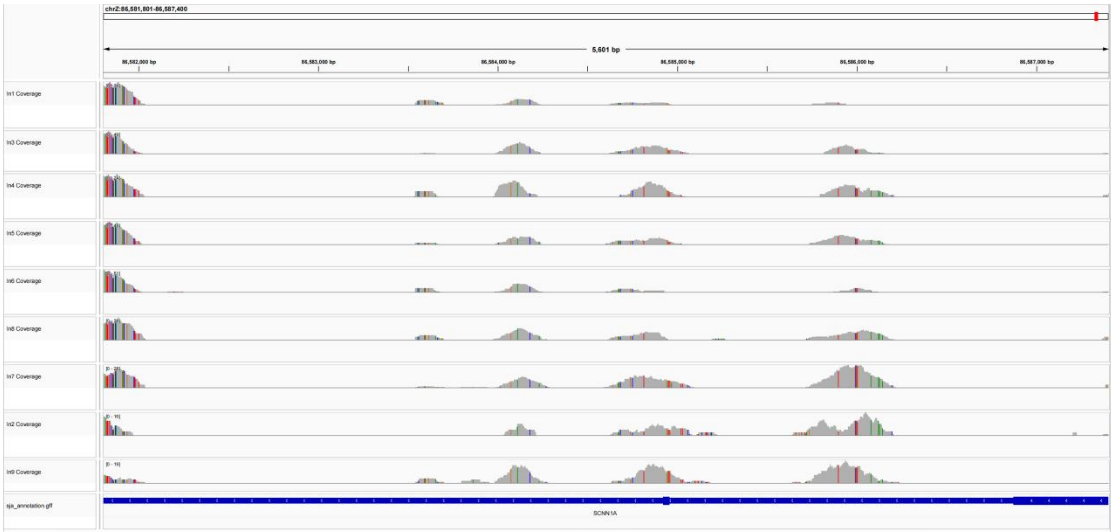

Figure S10. The coverage of DEL in the *SCNN1A* gene in Indonesia strain *of S. japonicum*, Related to Figure 3.

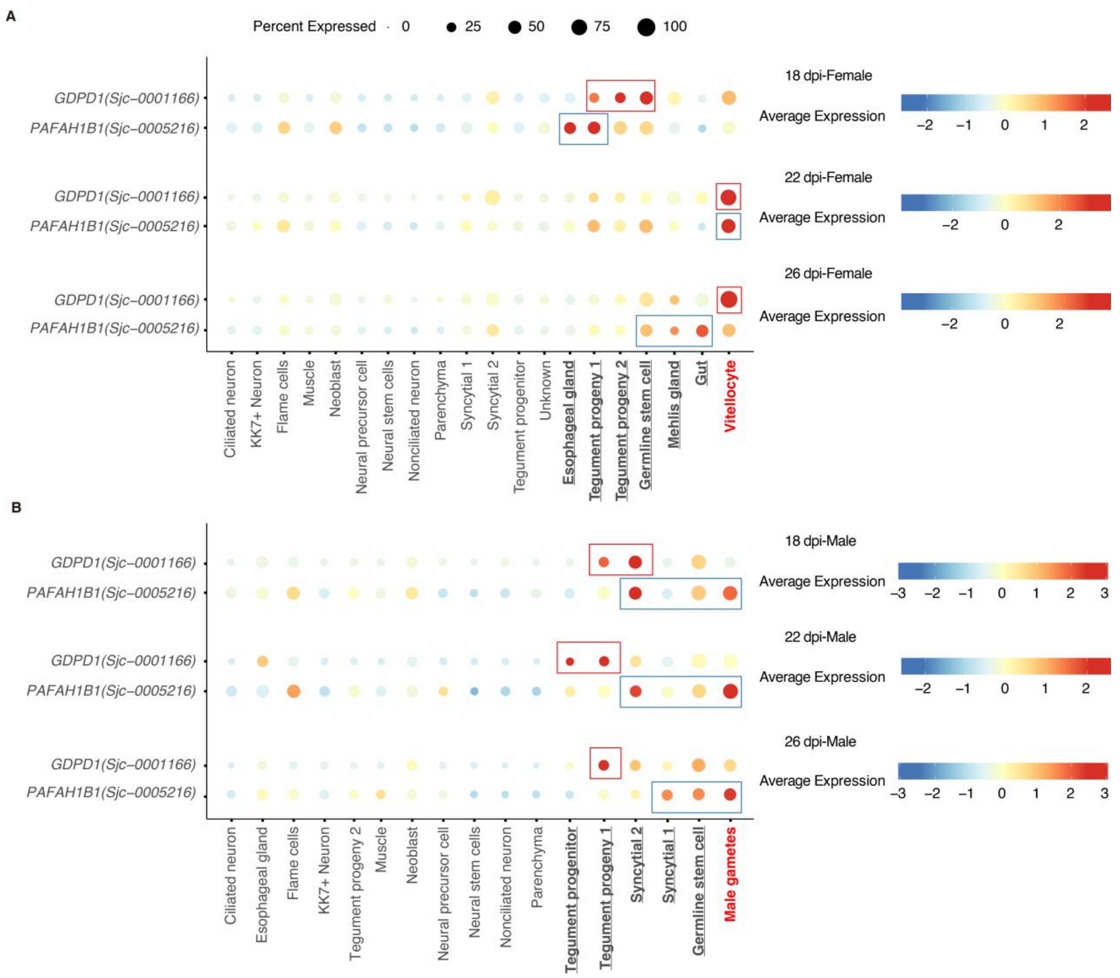

Figure S11. The *GDPD1* and *PAFAH1B1* genes for each cell type in *S. japonicum*, Related to Figure 5.

Dot plot displaying in the expression of *GDPD1* **(A)** and *PAFAH1B1* **(B)** genes for each cell type in *S. japonicum*. The cell types with red font were key reproductive organs in both male and femal *S. japonicum*. The cell types underlined were highly expressed types in male or female *S. japonicum*.
